# Cage-free eggs in China

**DOI:** 10.1093/af/vfac078

**Published:** 2023-02-23

**Authors:** Maria Chen, Huipin Lee, Yuchen Liu, Dan Weary

**Affiliations:** The Faculty of Land and Food Systems, The University of British Columbia, Animal Welfare Program, Vancouver, Canada; The Faculty of Land and Food Systems, The University of British Columbia, Animal Welfare Program, Vancouver, Canada; Shanghai University of Finance and Economics, Institute of Finance and Economics, Shanghai, China; The Faculty of Land and Food Systems, The University of British Columbia, Animal Welfare Program, Vancouver, Canada

**Implications**

- Some consumers in China prefer free-range products, but it is unclear if this preference is driven by a desire for improved hen welfare.
- Identification of cage-free eggs is difficult due to the low proportion of eggs being packaged and the mislabeling of products.
- Further research is needed to understand how cage-free systems in China can be best managed to improve hen welfare.

## Introduction

**Figure 1.**
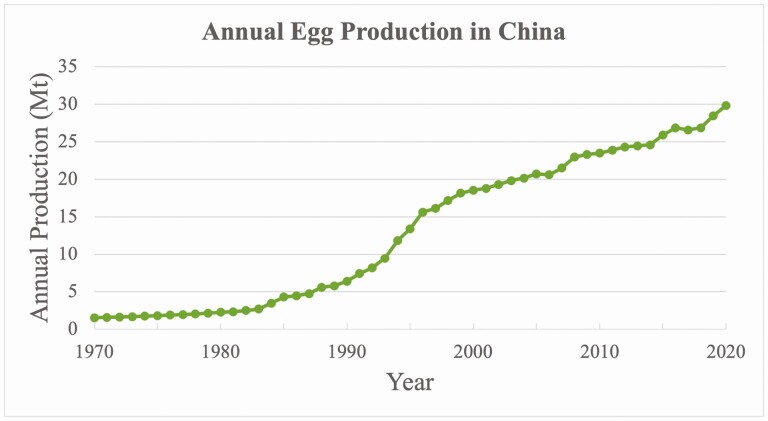
Annual egg production in China from 1969 to 2020, measured in million tons. Figure created using data from FAO (2022).

Since 1985, China has been the world’s largest egg producer. In 2020, 3.3 billion layer hens were reared in China, producing 29.8 million tons of eggs (around 40% of the world’s egg production, see Figure 1; FAO, 2022). Battery cage production became common in China in the 1980s and by 2017 approximately 90% of farms used cages (IEC, 2017).

Internationally, concern for animal welfare has begun to encourage a shift towards less confined housing systems, including cage-free housing for hens (with or without access to the outdoors). Any transition to cage-free systems will depend upon a variety of factors including the values of consumers and the way these eggs are marketed. The effects of this transition on hen welfare will depend on the degree to which cage-free housing methods actually benefit the birds themselves.

In this paper we review the development of cage-free farming in China in relation to consumer preferences and hen welfare. Our aims are to 1) describe the history of egg production in China, including changes in cage-free production, 2) describe current consumer preferences for eggs, including attributes associated with hen housing and welfare, 3) review the link between cage-free housing and hen welfare, 4) describe approaches to help improve hen welfare on cage-free farms, and 5) identify areas for future research.

## History of Cage-free Farming in China

After their domestication around 9,500 years ago (Wang et al. 2020), chickens became an important part of life in Chinese society, used for timekeeping, entertainment (e.g., cock-fighting), spiritual practices (e.g., sacrifice), and as a source of meat and eggs (Li, 2017). Chicken breeding and husbandry were well-documented in the agricultural text Essential Skills to Benefit the People (齐民要术; written around 540 CE). In the chapter devoted to chickens, recommended housing systems included indoor coops/cages with perches, sheltered coops with outdoor access, or bramble cages complete with nests provided in the walls (Jia, 2009).

In 1978, the government initiated the Reform and Opening Up policies to transition from a centrally planned to a mixed economy (Wang and Hu, 2010). As the economy grew and incomes rose, demand for animal protein increased. From 1979 to 2000, egg production increased from 2.1 Mt to 18.5 Mt, an almost 9-fold increase (see Figure 1; FAO, 2022).

After the 2000s, industry growth stabilized, and the industry began to focus more on food safety. This occurred in the context of major Avian Influenza outbreaks since 2001, food safety incidents of the 2000s, and the announcement of the completion of the poverty alleviation goals in 2020.

**Figure 2.**
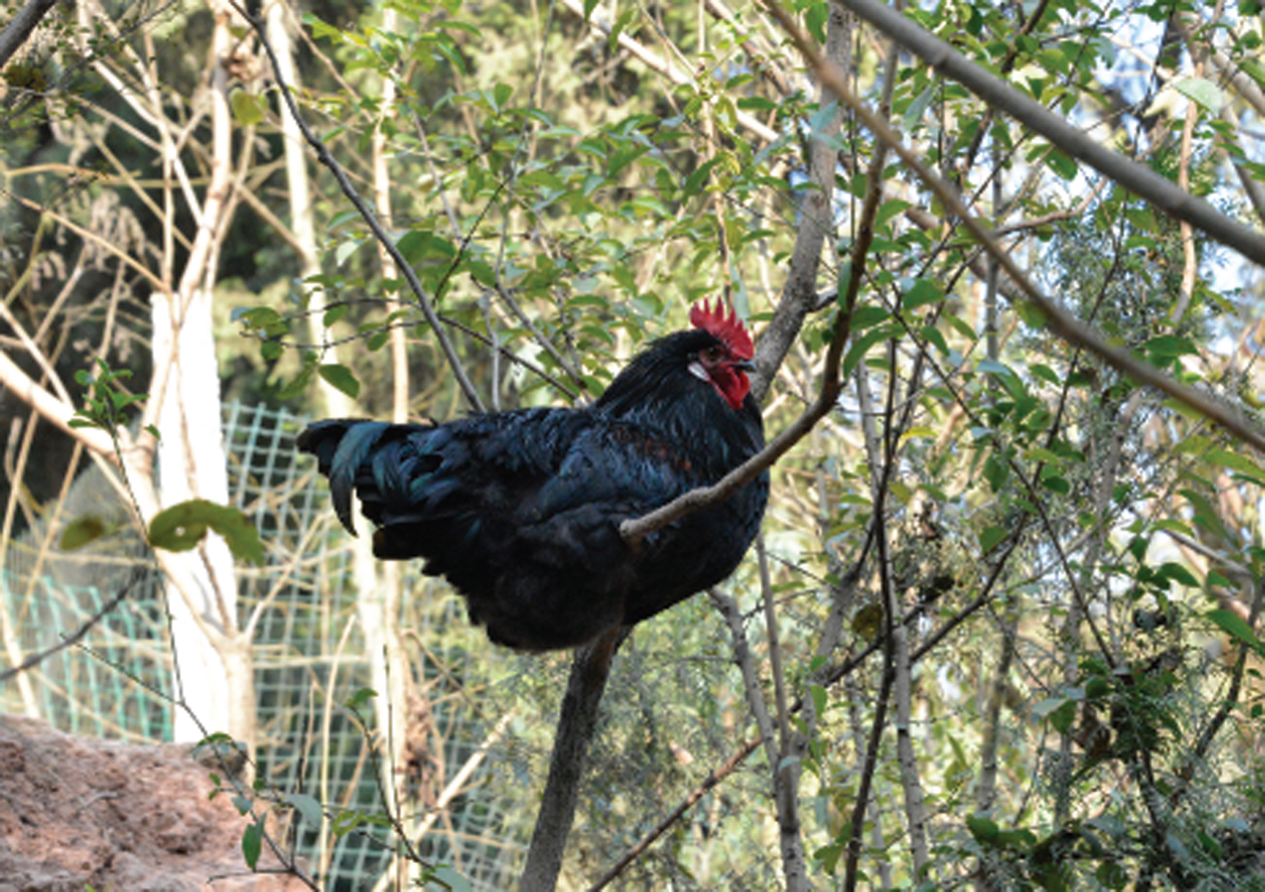
This Chishui black-bone chicken (赤水乌骨鸡; an indigenous breed) was seen perched on a branch on a farm from Xiangnong Ecological Agriculture Development Co. Ltd., winner of the 2018 Good Egg Production Award. Image used with permission, Xiangnong Ecological Agriculture Development Co., Ltd.

Today, diverse modes of production coexist, ranging from backyard, family farms to vertically integrated farms run by large companies (Yang, 2021), but most hens are housed in caged systems. Yang et al.’s (2014) survey of 18,909 farms in China, ranging in size from <2,000 to 500,000 hens, found that less than 2% of farms were cage-free, but smaller backyard farms (more likely to be cage-free) were likely underrepresented in this survey. The International Egg Commission estimated that 90% of farms in China used cages, while the remaining 10% were cage-free (with 9% being free-range and 1% being indoor only; IEC, 2017) (Figure 2).

There appears to be some demand for cage-free eggs (indoor and free-range) from retailers, food businesses, and consumers in China. More than 50 international food businesses and retailers have committed to sourcing cage-free eggs in China by 2025; food consultancy Lever China (2021) estimated that around 1.2 million eggs are needed each year to fulfill this commitment (0.0002% of China’s annual output of 604.7 billion eggs in 2020; FAO, 2022). Lever China also examined 873 packaged egg products sold in 10 major supermarkets in four first-tier cities in China and found that more than 17% of these products claimed to be produced in cage-free conditions (using descriptors such as “free-range” [散养], “cage-free” [非笼养], “organic”[有机], or images of cage-free environments).

## Consumer Preference for Egg Products

**Figure 3.**
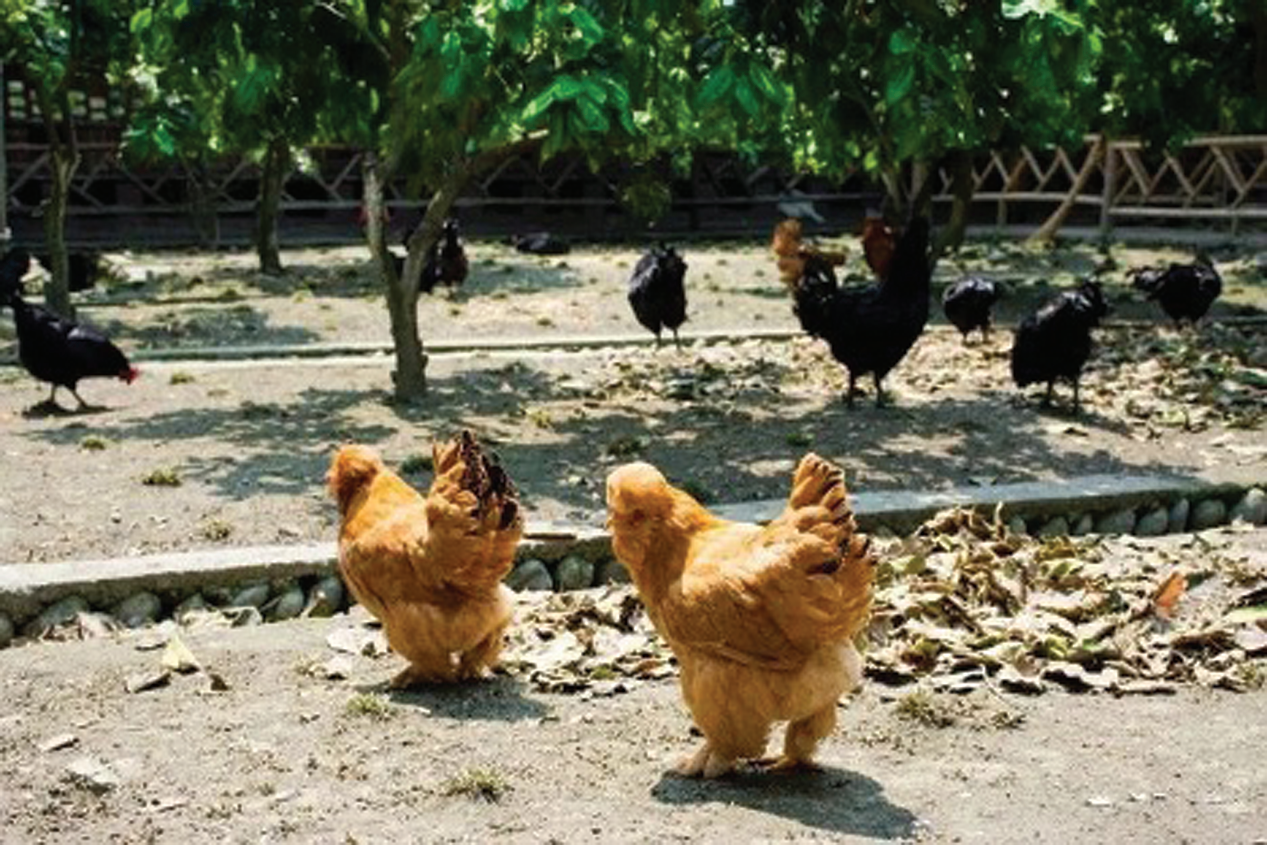
Beijing You Chicken (北京油鸡; a slow-growing, dual-purpose breed) in a free-range farm from Xiangnong Ecological Agriculture Development Co. Ltd., winner of the 2018 Good Egg Production Award. Image used with permission, Xiangnong Ecological Agriculture Development Co., Ltd.

While price is an important factor in purchasing decisions, consumers also consider other egg attributes. Readily identifiable characteristics such as egg size and color are important attributes to consumers during purchase; Yang (2021) reported that 55% of eggs purchased are brown, 43% are light brown, and the remainder are either white (1%) or blue (1%). Other attributes, such as production methods and welfare of the hens, are not readily identifiable to consumers and can only be inferred through packaging and marketing materials. Though fresh eggs sold at supermarkets and markets are sometimes packaged, false advertisements, and mislabeling can mean that identification of such attributes can be difficult. Some consumers in China indicate a preference for attributes such as nutritional value, food safety, considerations for the environment, and free-range. For example, Liu et al. (2022) found that consumers in Chongqing city have a preference for eggs labeled as free-range and show a willingness to pay a premium for these products; the authors also found that consumers with higher income, with members of the family who are pregnant or have children, and with higher levels of trust in and knowledge of labeling were more likely to choose eggs labeled as free-range. Bian and Liu (2014) conducted a survey to understand factors influencing egg purchasing behavior in Jiangsu province and found that nutrition and free-range were ranked as the top two factors influencing purchasing decisions, with other factors considered including packaging, egg size, egg color, price, service, and brand. The authors described consumer preference for “free-range” (散养) production and other commonly used terms including “indigenous chicken egg” (土鸡蛋) and “grass egg” (草鸡蛋) (which implies a more natural environment where chicken are able to forage for more natural foods rather than a commercial diet) (Figure 3).

The term “cage-free” (非笼养) is not commonly used by consumers in China, and to our knowledge, no peer-reviewed literature has examined consumer preferences for cage-free eggs per se. Consultancy firms IQC and FAI Farms surveyed consumers in 2020 and found that 40.5% of participants reported purchasing “free-range” eggs, but only 6.3% reported purchasing “cage-free” eggs, suggesting that these consumers were unaware of the overlap (The Poultry Site, 2022). Indeed, approximately 80% of participants reported being unsure of what “cage-free” eggs are and one-third reported having never hearing the term before. Thus, some people appear unfamiliar with the term “cage-free”, even though they may already be purchasing products they perceive as free-range.

There appears to be increasing awareness among the Chinese public of the term “animal welfare” (动物福利); in 2011, only a third of the Chinese public surveyed reported hearing of “animal welfare”, but by 2019, more than three-quarters of participants reported familiarity with this term (You et al., 2014; Liu et al., 2020). There is also evidence that Chinese consumers are willing to pay more for high welfare products; Wang et al. (2016) found that more than 80% of study participants were willing to pay more for high welfare pork products.

Though the terms “cage-free” and “animal welfare” were both translated from English, cage-free production and concepts related to a good life for animals (e.g., unity between man and nature, and compassion for animals; Cao, 2020) are deeply rooted in Chinese culture and history. Further research should identify engagement strategies and appropriate messaging which leverage this cultural background.

## Animal Welfare in Cage-free Systems

Housing methods can profoundly affect animal welfare, but the effects vary depending upon the housing used and the way “animal welfare” is conceived. One commonly used conception of animal welfare encompasses the animals’ health, affective state, and ability to express natural behaviors (Fraser et al., 1997). Conventional cages are often favored by egg producers due to ease of management, lower cost of production, and increased hygiene (Duncan, 2001). Less confined housing systems provide potential benefits for hens, including the ability to express more of their full behavioral repertoire. When appropriate resources are provided (e.g., litter, perch, nest; Hartcher and Jones, 2017) hens are able to perform highly motivated behaviors like foraging, dustbathing, perching, and nesting (Hemsworth and Edwards, 2020). At the same time, cage-free systems can be associated with poor hen health (especially in poorly managed litter systems; Duncan, 2001), injurious feather pecking potentially leading to cannibalism (especially at high stocking densities and without proper foraging opportunities; Lambton et al., 2010), and fractures during laying period (especially with poor housing design; Hartcher and Jones, 2017).

Improvements to animal welfare on cage-free farms require both producer support and appropriate management. Yang (2020) interviewed egg producers in China using conventional caged systems and found that perceived barriers for converting to cage-free farming include perceived consumers’ unwillingness to pay, the absence of trustworthy food labeling, lack of land, restrictions related to environmental policies, and challenges to managing cage-free systems. Addressing these concerns may encourage producers to adopt cage-free systems, however, appropriate management is still required to ensure animal welfare on cage-free farms. Strategies to address common animal welfare concerns in cage-free systems include improved biosecurity, environmental and litter management, vaccination, and monitoring (McMullin, 2022). Some producers try to reduce feather pecking through beak trimming and lowering light levels, but the former causes pain and the latter can lead to abnormal eye development. Feather pecking may also be reduced by carefully managing stocking density and by providing rewarding foraging opportunities (Lambton et al. 2010). Better perch design and placement can help reduce fractures during laying period (Hartcher and Jones, 2017).

Genetics can influence a hen’s ability to thrive in cage-free environments. For example, Leenstra et al. (2016) found less feather pecking in white leghorn hens compared to brown hens when these birds were housed in cage-free systems. China has many breeds of laying hens, including more than 100 indigenous breeds and an increasing number of commercial breeds including the Wuhua chicken (with improved disease resistance; Weng et al., 2020) and the Niya chicken (with improved heat resistance; Gu et al., 2020). To our knowledge, no research on chicken genetics in China has focused on hen welfare, or what breeds thrive under cage-free conditions.

**Figure 4.**
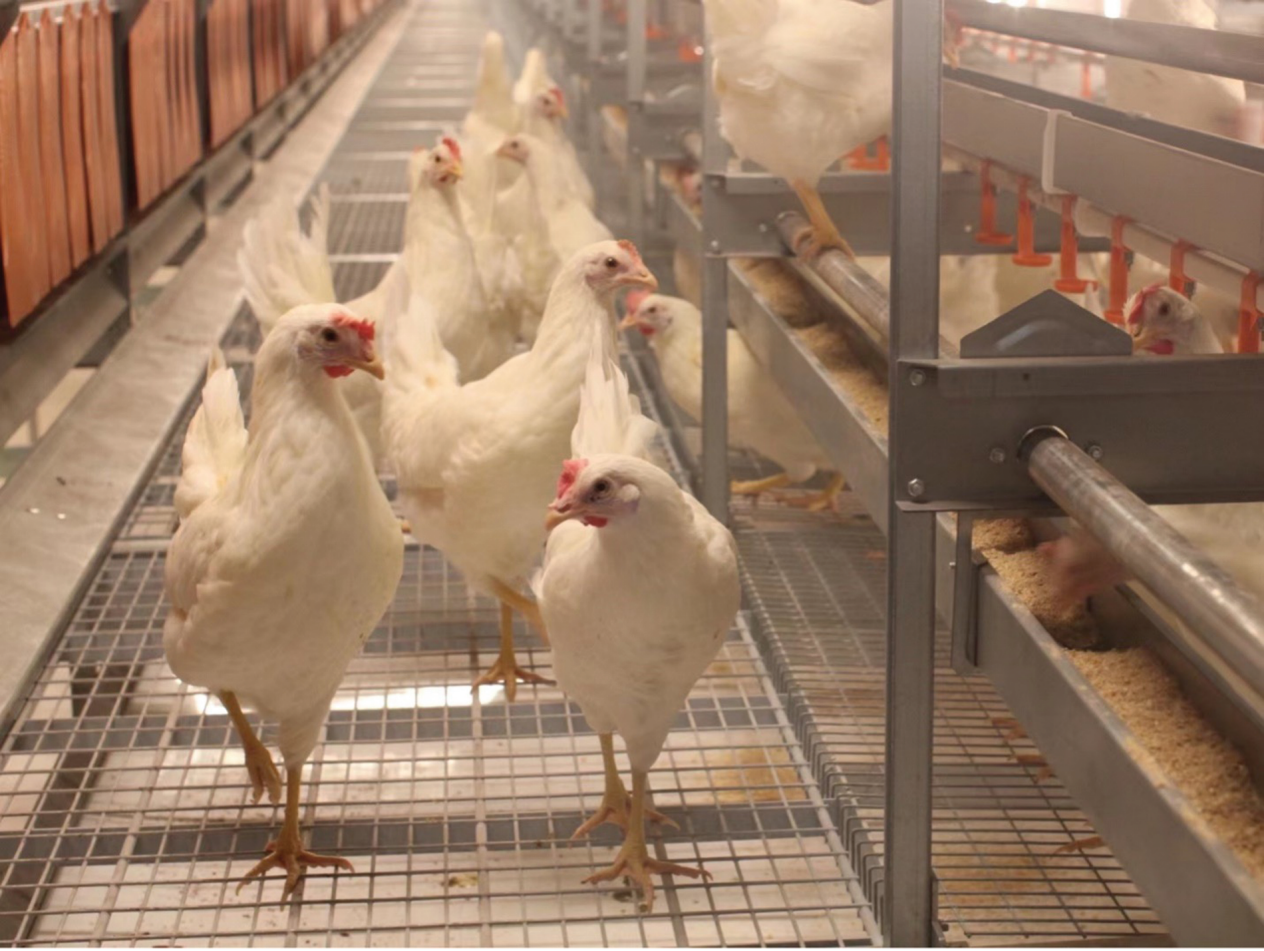
Hy-line white layer hens (a highly productive, foreign commercial breed) reared in indoor, multi-tier system on a farm in China. Image used with permission, Happy Eggs (Hainan) Agriculture Development Co., Ltd.

Overall, cage-free housing refers only to a lack of caged systems, and the welfare of the hens is dependent on other important factors such as housing design, management, and use of appropriate hen breeds (Figure 4).

## Approaches to Help Assure Hen Welfare on Cage-free Farms

Cage-free housing does not guarantee higher hen welfare, but a variety of strategies are available to encourage higher animal welfare on farms within China and these can apply to cage-free farms. Though there are no laws and regulations focused on farm animal welfare or cage-free production, there are various voluntary standards, corporate assurance programs, producer awards, and product differentiation or labeling schemes which may help improve hen welfare on cage-free farms.

Though not explicitly mentioning “animal welfare” or “cage-free”, various regulations at the national and regional levels affect the welfare of laying hens, including those relating to animal husbandry, transport, and slaughter. Examples of national laws include the “Animal Husbandry Law” (including requirements for livestock breeding, husbandry, farm equipment, staffing, waste management, and transport) and the “Animal Epidemic Prevention Law” (focused on animal health and the prevention and control of epidemics). Examples of relevant administrative regulations include the “Regulations on the Administration of Feed and Feed Additives”, “Regulations on the Administration of Veterinary Drugs”, and “Regulations on the Prevention and Control of Pollution from Large-scale Livestock and Poultry Breeding”.

International trade agreements have been suggested as a strategy to improve animal welfare, but this mechanism would likely have limited effect given that China is self-sufficient in egg production (with low levels of import and export of eggs; Yang et al., 2018). China has been part of the World Trade Organization (WTO) since 2001, and as such follows the World Organization for Animal Health’s (WOAH) “Terrestrial Animal Health Codes” (last updated in English in 2020 and translated into Mandarin Chinese in 2021). This nonmandatory international standard provides guidelines to improve animal health and welfare of layer hens (including husbandry, transport, slaughter, and culling).

There are two industry-formulated standards that cover cage-free egg production in China: the “Farm Animal Welfare Requirements: Layer Hens” in 2017, and the “Evaluation Guidelines of Cage-free Egg Production” in 2021. The 2017 standard covers animal welfare in both caged and cage-free systems, while the 2021 standard focuses solely on cage-free systems. The 2017 standard was developed by China Association for Standardization in collaboration with China’s only farm animal welfare organization (the International Cooperation Committee for Animal Welfare; ICCAW), in addition to academics, experts, and industry. The 2021 standard was developed by China Chain Store and Franchising Association (CCFA) in collaboration with consultancy firms, industry, and other organizations. Both sets of guidelines describe recommended practices for egg producers, including the standardization and management of pullet sourcing, food safety, environment, feed and water, husbandry, health, and culling.

Corporate quality assurance programs can help motivate farmers to adopt certain practices, including cage-free production systems. More than 50 international companies with operations in China have committed to sourcing only cage-free eggs by 2025, but to our knowledge few have reported progress towards this goal. According to tracking by the organization Chicken Watch (2022), only 8 of these companies currently report sourcing all their eggs in China from cage-free farms.

There are two producer awards for farms committed to practices associated with good hen welfare: the Good Egg Production Award, and the Mingteyouxin Award (名特优新; Famous, Special, Excellent, and New Award). The Good Egg Production Award in an award established in by Compassion in World Farming (CIWF), with the first recipients in China in 2017. To date, 17 producers have been awarded the Good Egg Production Award, recognizing their current use or commitment to use cage-free systems within five years, with a rating of one to five stars depending on additional animal welfare criteria met or committed to. The Mingteyouxin Award is an award approved and audited by the Ministry of Agriculture and Rural Affairs (MARA) to recognize outstanding agricultural products. Since 2020, layer farms that meet the “Farm Animal Welfare Requirements: Layer Hens” can be recommended and audited by the ICCAW for further approval and auditing by the MARA. To date, MARA has awarded 7 egg producers the Mingteyouxin Award.

**Figure 5.**
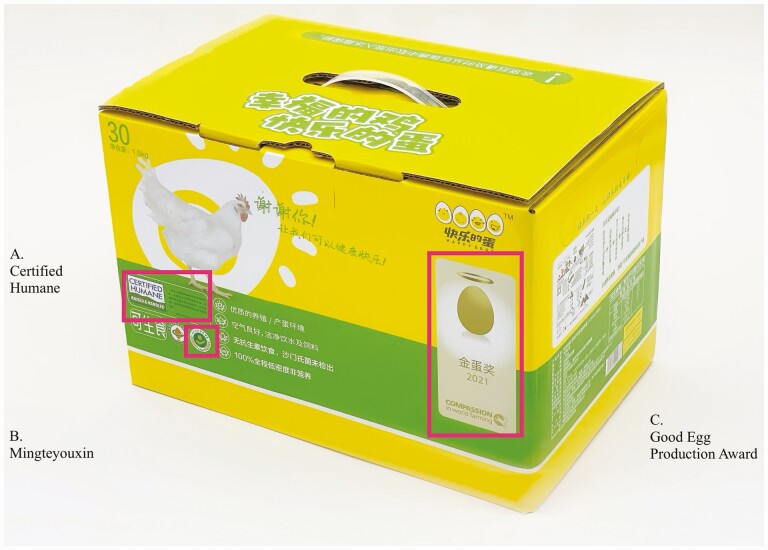
Egg packaging from Happy Egg, labeled to show one certification (A. HFAC’s Certified Humane) and two awards (B. MARA’s Mingteyouxin Award and C. CIWF’s Good Egg Production Award). Image used with permission, Happy Eggs (Hainan) Agriculture Development Co., Ltd.

The purchasing decisions of consumers could also help motivate producers to adopt high welfare practices. One limitation of this approach is that it can be difficult for consumers to identify eggs produced to a higher welfare standard or in specific housing systems. Yang (2011) estimates that less than 10% of table eggs are packaged, and that branding is not always accurate, such that some eggs produced in cage-free conditions may not be identified as such, and eggs branded as cage-free may in fact be from caged systems. Labeling schemes can facilitate consumer identification of specific egg attributes. In 2021, the animal welfare organization Humane Farm Animal Care (HFAC) launched the Certified Humane certification program in China. The certification program requires third-party auditing based on the HFAC standards, covering feed and water, environment, management (e.g., monitoring, working understanding of animal welfare), health, euthanasia, transport, and culling. To date, 8 cage-free farms have received the Certified Humane certification, and these products are available both online and offline in major Chinese cities (HFAC, 2022) (Figure 5).

## Future Directions

This paper provides an overview of developments in cage-free eggs in relation to consumer preference and hen welfare in China, based on the limited academic and grey literature available on this topic. Our review suggests that further research is still required on a number of related topics.

Firstly, work is required to better understand consumers in China, including what they value and what messaging strategies they are most receptive to. In particular we recommend research on consumer preference, values, and behaviors associated with cage-free eggs and higher animal welfare products, including qualitative studies designed to better understand motivations behind purchasing behaviors. For example, it is important to understand what cage-free and animal welfare mean to consumers, and to what extend interest in cage-free products is associated with an interest in animal welfare and other values. As consumers are diverse in their preferences and values, further research is needed to identify characteristics of consumers who are most receptive to cage-free eggs and high welfare products, helping to identify key influencers and appropriate messaging, marketing channels, branding, food labeling, and placement of products.

Secondly, we call for research identifying welfare challenges and potential solutions for cage-free farms in China. Given the diverse conditions on cage-free farms in China (including variable geography, climate, ecology, breed, housing design and equipment, management), approaches to addressing animal welfare issues will need to be tailored to specific contexts. Research is needed to understand the welfare conditions on Chinese farms, focusing on resource-based measures (such as housing design), management-based measures, and animal-based measures (which more directly assess the conditions and experience of animals). Given the importance of management and stockmanship, we recommend research into strategies to engage farm workers and encourage better animal welfare practices. We also call for research on how genetics of layer hens can affect welfare (e.g., identifying breeds that thrive under cage-free rearing conditions); this topic is particularly important because of the rich diversity of indigenous breeds in China.

Finally, we call for new work to examine the effectiveness of different strategies for improving hen welfare on cage-free farms. Various quality assurance schemes have been proposed with relevant requirements for hen welfare, but further work is now needed to assess how these translate to living conditions on farms, and how these programs can be implemented more effectively (e.g., through regular audits of animal-based outcomes). There is also a need to understand the efficacy of different approaches to informing consumers about egg attributes which are not otherwise easily identifiable (including production method and animal welfare). Specifically for product differentiation schemes, research is needed to explore consumer knowledge and trust in labels, and in the organizations providing the information. Future studies can also examine alternative ways for consumers to learn about egg products, for example through direct communication between consumer and producers at markets and online social platforms.

## Conclusions

Traditionally, egg production in China has occurred on small, cage-free farms, but caged systems now represent approximately 90% of production. Cage-free production has the potential to achieve higher animal welfare compared to caged systems, for example by providing more room for hens to move and express natural behaviors, but cage-free farms do not guarantee higher hen welfare, and new research and policy efforts are required to ensure that hens benefit from these systems.

Chinese consumers express a preference for “free-range” products, and products associated with free-range housing. To some extent, this preference may also be related to interest in animal welfare but this link is still uncertain. In a context where higher welfare products are not easily accessible and identifiable to consumers, eggs identified as cage-free provide at least some opportunity for consumers to choose products potentially associated with higher welfare, and may even increase awareness of farm animal welfare issues more generally. We suggest that the topic of cage-free eggs provides helpful lessons for those who seek to promote other products in China intended to improve farm animal welfare.

## About the Authors

Maria Chen is a Research Technician at the Animal Welfare Program in the University of British Columbia, and the Cofounder of the International Farm Animal Welfare Fellowship. She graduated her MSc and BSc degrees in Applied Animal Biology at the University of British Columbia. Her research interests include understanding how to address animal welfare issues in culturally compelling ways which work for both human and non-human animals. She uses qualitative research (mainly ethnography involving immersive fieldwork) to understand the lives and experiences of her participants. She has researched laboratory users’ perceptions of rats in Canada, Karen mahouts’ (elephant guardians) relationships with Asian elephants in Thailand, employee management and animal care on dairy farms in China, and the sale of cage-free eggs in China.

Huipin (Julia) Lee is a Graduate Researcher at the Animal Welfare Program in the University of British Columbia, and a Freelance Researcher and Consultant at FAI Farms. She graduated from her BVM at the Royal Veterinary College. Her research focuses on understanding the cultural context of human-animal interfaces through exploring the perspectives of people involved in activities impacting animals. Through semi-structured interviews, she has addressed ethical concerns of researchers conducting research on working equids in low- and middle-income countries. She is currently conducting an online ethnography with cage-free egg consumers in China, which would shed light on both their perspectives and behaviours of purchasing and consuming cage-free eggs.

Liu Yuchen is a PhD student at the Institute of Finance and Economics, Shanghai University of Finance and Economics. His research interest includes exploring the impact of food labels (such as animal welfare) on consumer behavior from an economic perspective. Qualitative and quantitative methods were applied in his study to analyze the benefits of animal welfare to the high-quality development of animal husbandry. At present, his research focuses on consumers’ cognition and demands of farm animal welfare (including pigs, broilers, laying hens).

Dan Weary is a Professor at The University of British Columbia. Dan did his BSc and MSc at McGill and DPhil at Oxford before co-founding UBC’s Animal Welfare Program where he now co-directs this active research group. Dan’s research focuses on developing measures for the assessment of animal welfare and developing practical methods of improving the lives of animals. Dan’s animal research has helped lead to the changes in management practices (including the adoption of higher milk rations for calves and pain management for disbudding) and housing methods (including the adoption of pair and small group rearing for pre-weaned calves). Dan has also led research on attitudes regarding animal care practices, with the aim of identifying areas of shared concern as well as barriers to adopting practices that can improve the lives of animals.
