# Correction to: Cage-free eggs in China

**DOI:** 10.1093/af/vfad018

**Published:** 2023-04-15

This is a correction to: Maria Chen, Huipin Lee, Yuchen Liu, Dan Weary, Cage-free eggs in China, , Volume 13, Issue 1, February 2023, Pages 34–39, https://doi.org/10.1093/af/vfac078

In the originally published version of this manuscript, there was a typographical error in the third author’s name. This should read: “Yuchen Liu” instead of “Yuchen Lin”.

This error has been corrected within the article.

